# Supplementary material for: Sexual dimorphic metabolic and cognitive responses of C57BL/6 mice to Fisetin or Dasatinib and quercetin cocktail oral treatment
Source: GeroScience. 2023 Jun 9;45(5):2835–50. doi: 10.1007/s11357-023-00843-0 (PMC10643448; doi:10.1007/s11357-023-00843-0)
Supplement: Supplementary file 1 — Supplementary file1 (DOCX 738 KB) [file 11357_2023_843_MOESM1_ESM.docx]

**Supplemental Information: Figure 1: Effects of senotherapeutic treatment on the hippocampal SASP profile in C57BL/6 mice.** mRNA expression levels of hippocampal SASP markers (a-f) were measured at time of euthanization after 10 treatments. Data are presented as means ± SEM (n = 8-12). A two-way ANOVA was used to determine P-values for the categorial variables (S = Sex and T = Treatment) and their interaction (S x T), which are shown for each bar graph. *p < 0.05, **p < 0.01, ***p<0.001 based on a two-tailed Student's t test.

**Supplemental Information Figure 2: Senotherapeutic treatment altered plasma cytokine concentrations in C57BL/6 mice.** Plasma concentrations of TNFα, MCP1, IL-6, and IL-10 (a-d) from time of euthanization after 10 senotherapeutic treatments. Data are represented as means ± SEM (n = 8). A two-way ANOVA was used to determine P-values for the categorial variables (S = Sex and T = Treatment) and their interaction (S x T), which are shown for each bar graph. *p < 0.05, **p < 0.01, ***p<0.001 based on a two-tailed Student's t test.

**Supplemental Information Figure 3: Effects of senotherapeutic treatment on physical performance in C57BL/6 mice.** MWM swimming speed during the training days and probe challenge (a-d) and forepaw grip strength (e). Data are presented as means ± SEM (n = 16-20). A two-way ANOVA was used to determine P-values for the categorial variables (S = Sex and T = Treatment) and their interaction (S x T), which are shown for each bar graph.

**Supplemental Information Figure 4: Hippocampal mRNA expression of genes involved with synaptic plasticity were unaltered after senotherapeutic treatment.** Hippocampal mRNA expression from time of euthanization after 10 senotherapeutic treatments in C57BL/6 mice. Data are represented as means ± SEM (n = 16-20). A two-way ANOVA was used to determine P-values for the categorial variables (S = Sex and T = Treatment) and their interaction (S x T), which are shown for each bar graph. **p < 0.01 based on a two-tailed Student's t test.

**Supplemental Information Figure 5: Effects of aging on physical, metabolic, and cognitive parameters.** Nontreated four-month-old C57BL/6 mice were compared to sex-matched control treated mice. Data are represented as means ± SEM (n = 16-20). A two-way ANOVA was used to determine P-values for the categorial variables (S = Sex and A = Age) and their interaction (S x A), which are shown for each bar graph. *p<0.05, **p < 0.01, ***p<0.001 based on a two-tailed Student's t test.

**Supplemental Information Figure 6: Distance Traveled (centimeter) during** **acclimation day in open field of Novel Object Recognition (NOR) test.** Data are presented as means ± SEM (n = 16-20). A two-way ANOVA was used to determine P-values for the categorial variables (S = Sex and T = Treatment) and their interaction (S x T), which are shown for each bar graph. **p < 0.01, ***p < 0.001 based on a two-tailed Student's t test.

**Supplemental Information: Table 1:** A list of forward and reverse primers used in this study.

**Supplemental Information: Figure 1**

**
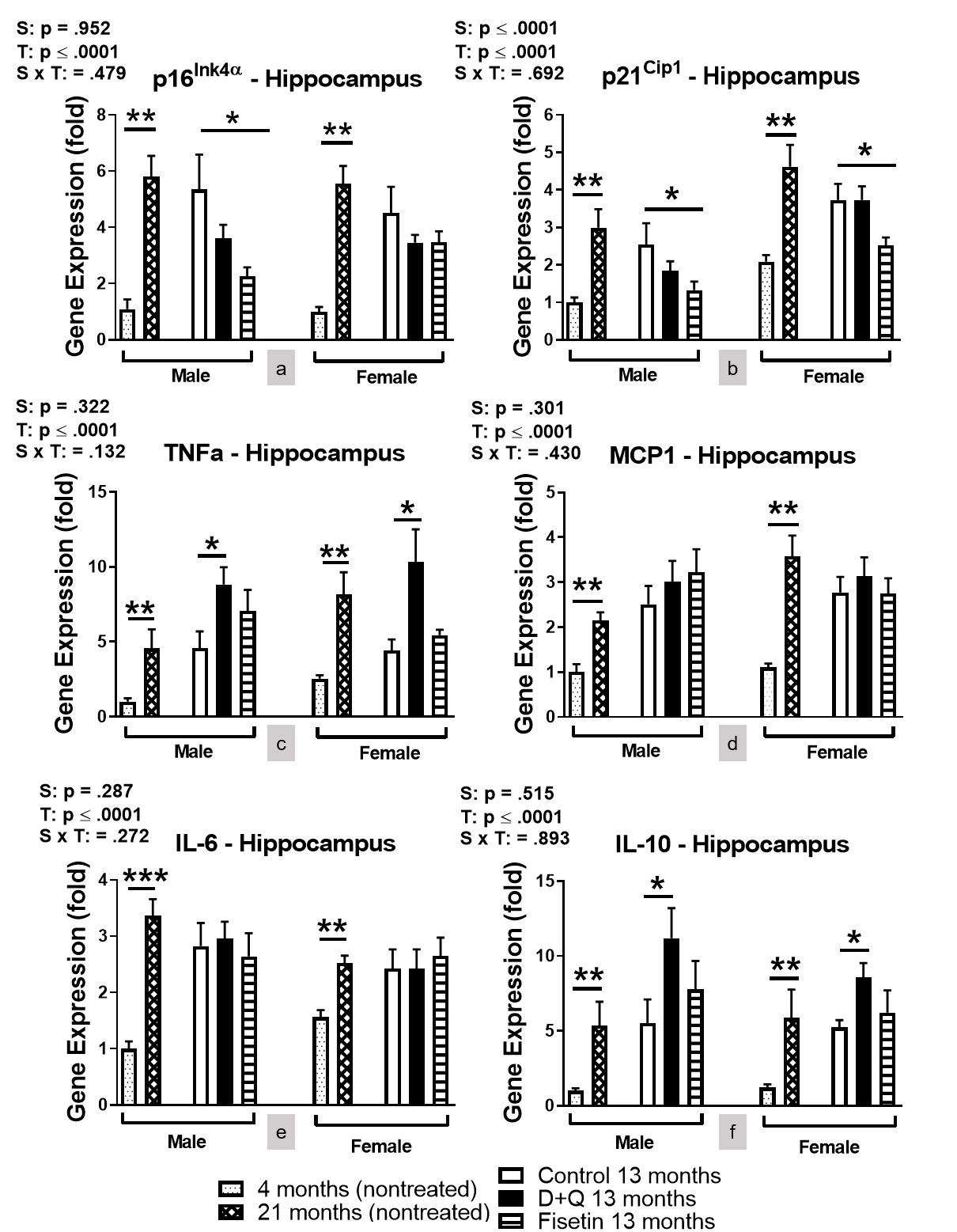
**

**Supplemental Information: Figure 2**

**
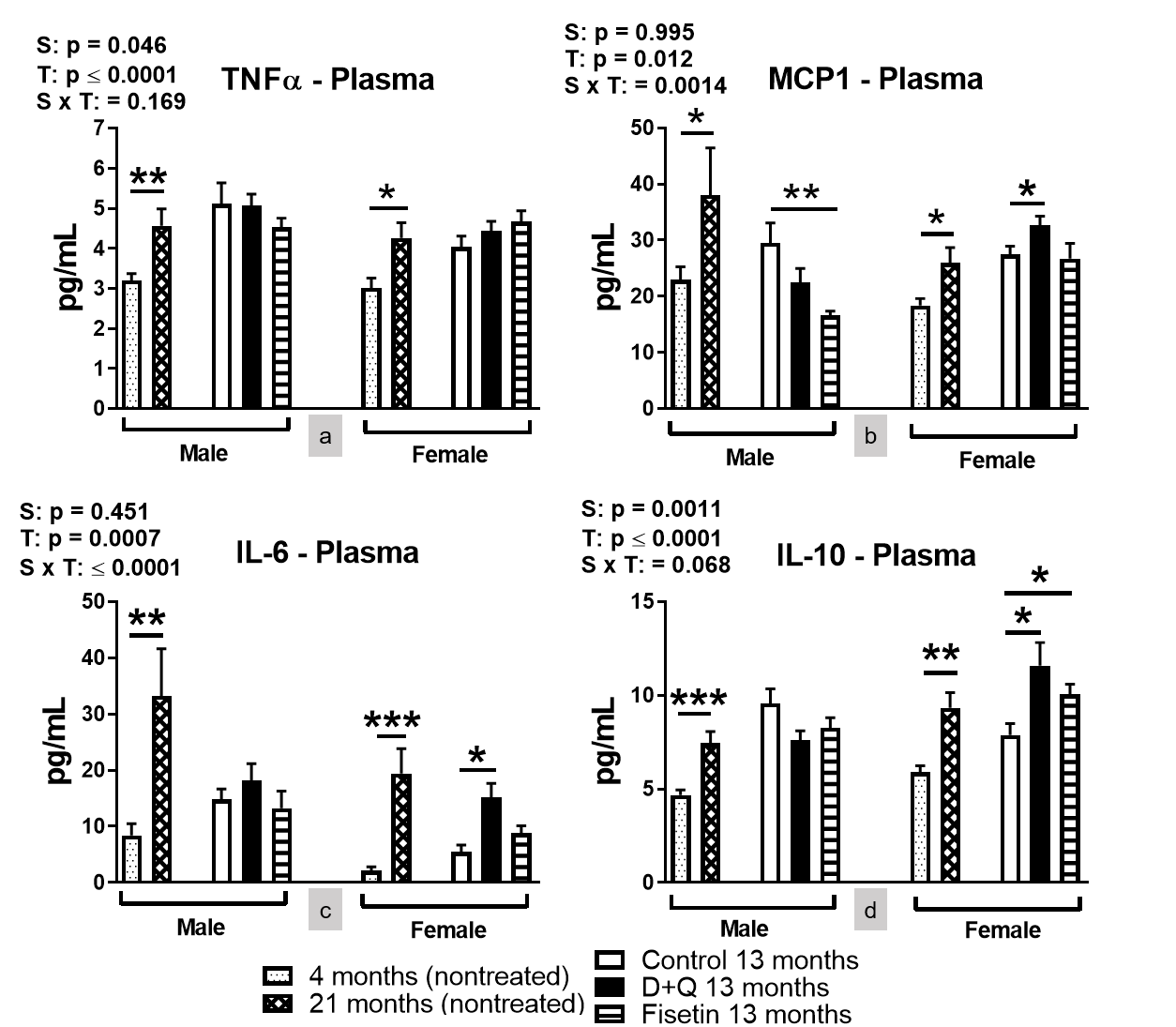
**

**Supplemental Information: Figure 3**

**
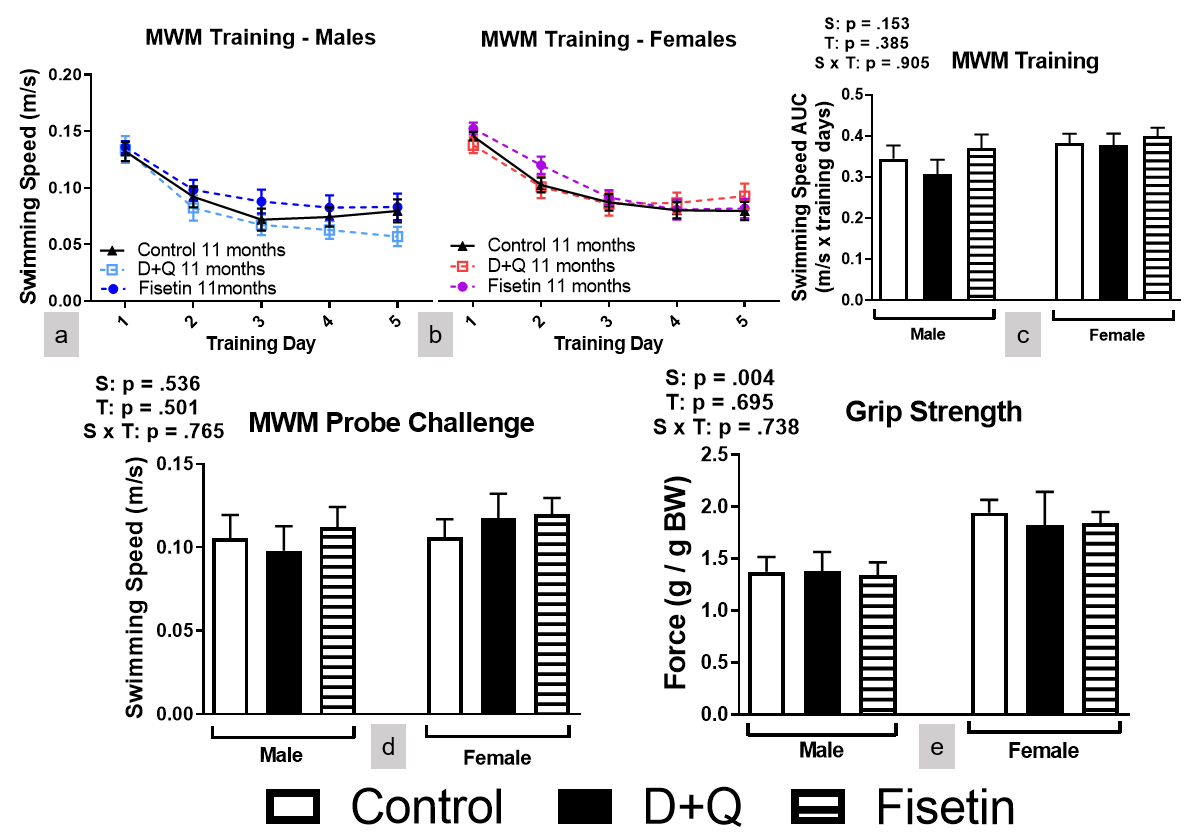
**

**Supplemental Information: Figure 4**

**
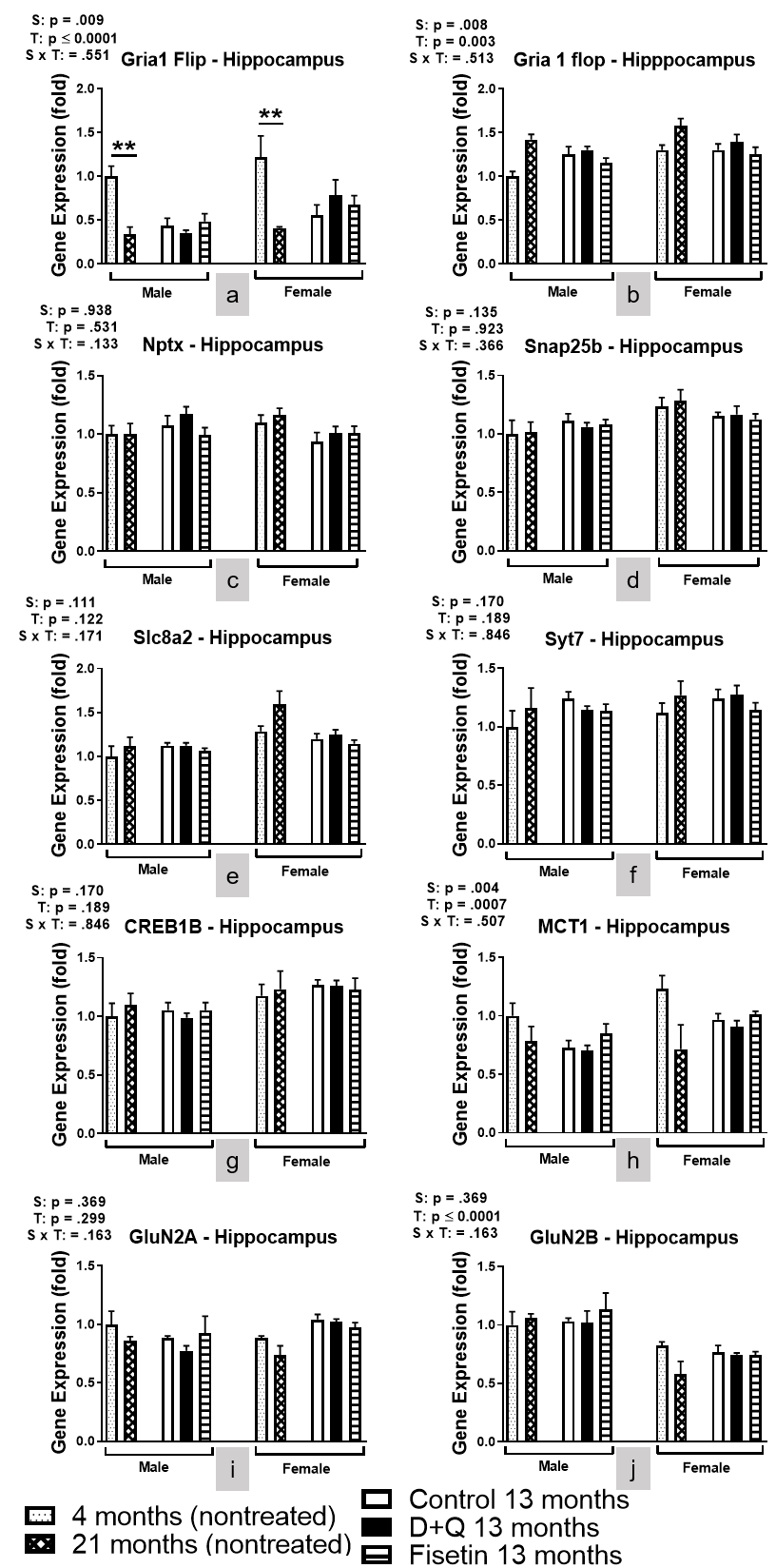
**

**Supplemental Information: Figure 5**

**
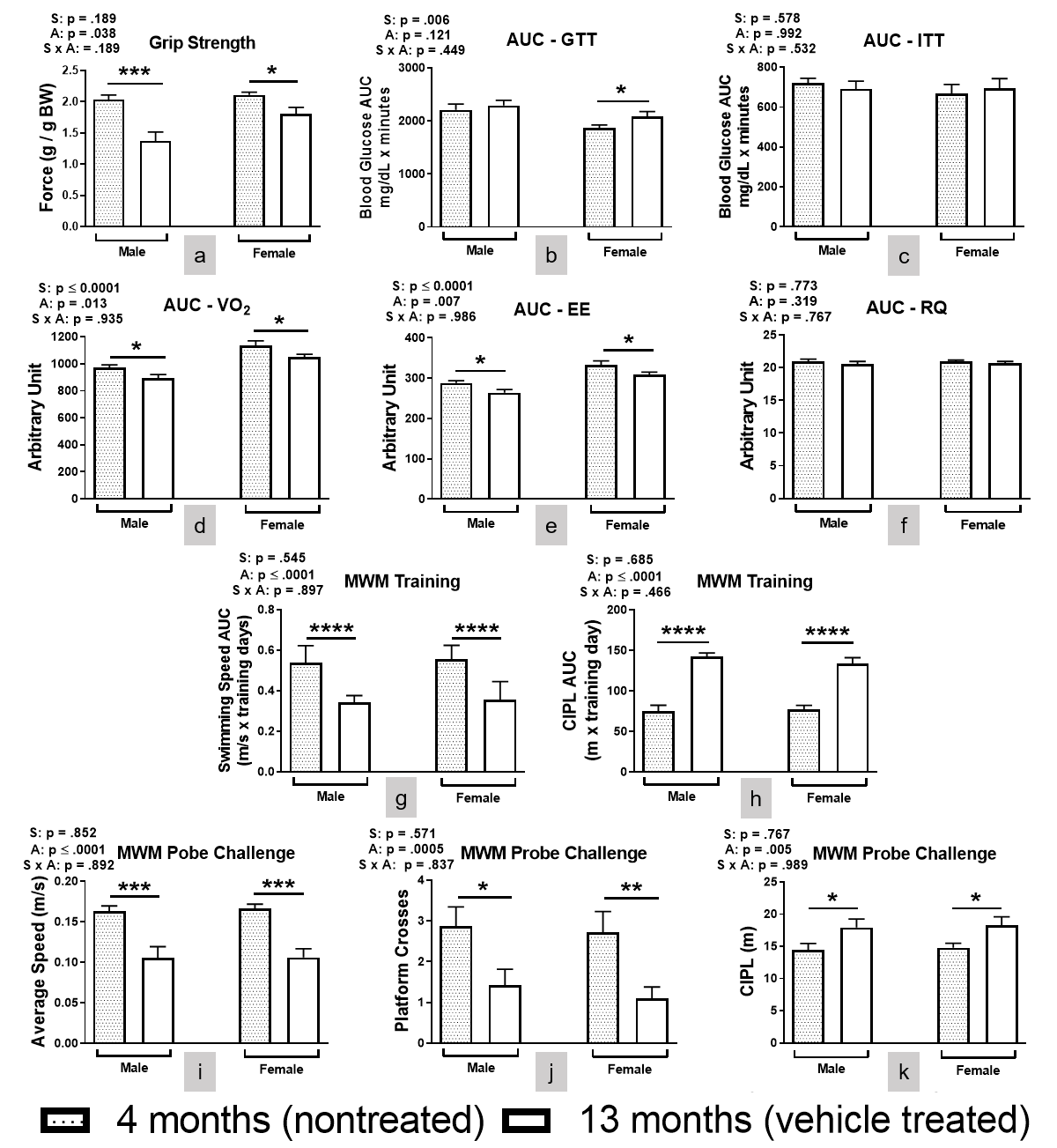
**

**Supplemental Information: Figure 6**

**
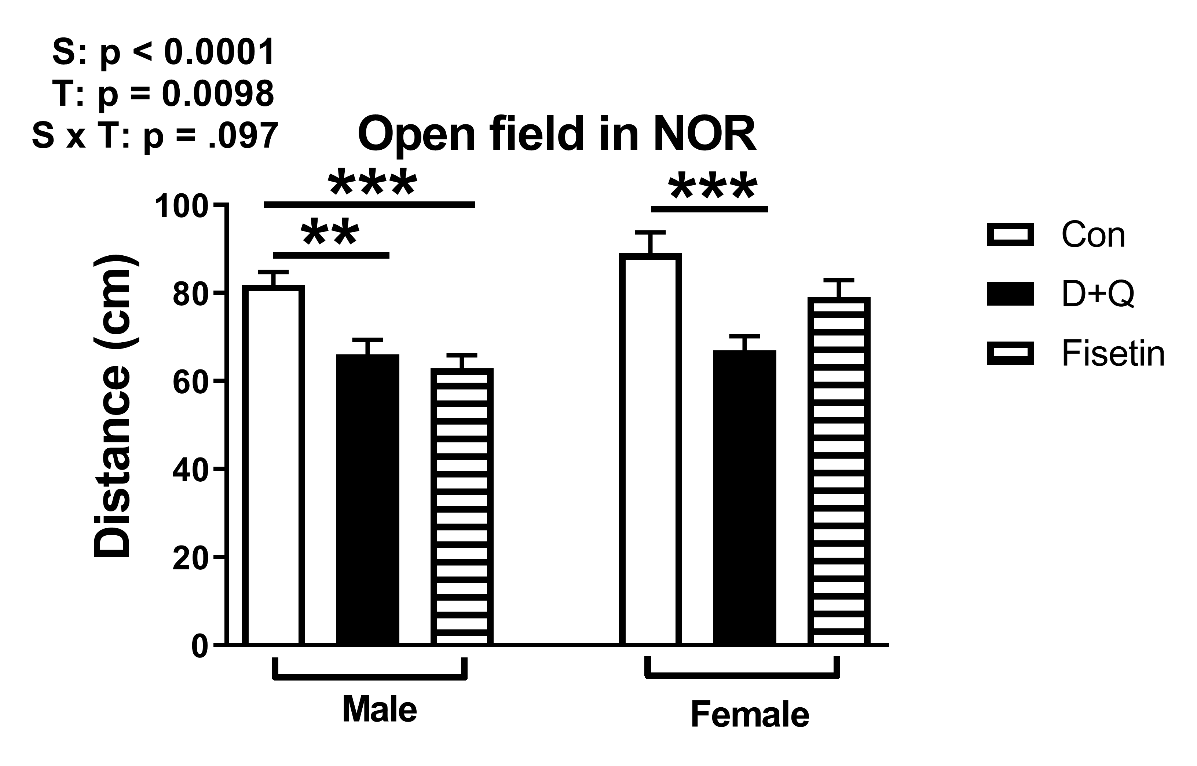
**

**Supplemental Information: Table 1**

|  |  |  |  |  |  |
| --- | --- | --- | --- | --- | --- |
|  | **Gene** | **Protein** | **Forward** | **Reverse** |  |
|  | CDKN2A | p16^Ink4α^ | TACCCCGATTCAGGTGAT | TTGAGCAGAAGAGCTGCTACGT |  |
|  | CDKN1A | p21^Cip1^ | CCTGGTGATGTCCGACCTG | CCATGAGCGCATCGCAATC |  |
|  | TNFα | TNFα | GCCTCTTCTCATTCCTGCTTG | CTGATGAGAGGGAGGCCATT |  |
|  | CCL2 | MCP1 | CCACTCACCTGCTGCTACTCAT | TGGTGATCCTCTTGTAGCTCTCC |  |
|  | IL6 | IL-6 | TAGTCCTTCCTACCCCAATTTCC | TTGGTCCTTAGCCACTCCTTC |  |
|  | IL10 | IL-10 | GCTCTTACTGACTGGCATGAG | CGCAGCTCTAGGAGCATGTG |  |
|  | ACACA | ACC | ATGGGCGGAATGGTCTCTTTC | TGGGGACCTTGTCTTCATCAT |  |
|  | UCP1 | UCP1 | AGGCTTCCAGTACCATTAGGT | CTGAGTGAGGCAAAGCTGATTT |  |
|  | PPARG | PPARγ | ACCCCCTGCTCCAGGAGAT | TGCAATCAATAGAAGGAACACGT |  |
|  | NPTX | Nptx | ACACCATGAAAGTGGGAGGTAACT | ACTGGTCTTGTCCTTACTTCCGGA |  |
|  | SNAP25 | Snap25b | CTCATCGAGTGGGGCAACA | GTGATGCCATTTGCCATCGTT |  |
|  | Syt7 | Syt7 | ACTCCATCATCGTGAACATCATC | TATGTCGAAGGCGAAAGAC |  |
|  | Slc8a2 | Na^+^ / Ca^++^ Exchanger, Member 2 | AACAGCACCTTCTACGTGG | GTCCTTCTGCGTCTCCC |  |
|  | GRIN2A | GluN2A | ACGTGACAGAACGCGAACTT | TCAGTGCGGTTCATCAATAACG |  |
|  | GRIN2B | GluN2B | CAGCAAAGCTCGTTCCCAAAA | GTCAGTCTCGTTCATGGCTAC |  |
|  | CREB1 | Creb1b | AGCAGCTCATGCAACATCATC | AGTCCTTACAGGAAGACTGAACT |  |
|  | SLC16A1 | MCT1 | TGTTAGTCGGAGCCTTCATTTC | CACTGGTCGTTGCACTGAATA |  |
|  | GRIA1 | Gria1 Flip | ACACCATGAAAGTGGGAGGTAACT | ACTGGTCTTGTCCTTACTTCCGGA |  |
|  | GRIA1 | Gria1 Flop | GTCCGCCCTGAGAAATCCA | GCACTCGCCCTTGTCGTA |  |
|  | SLC2A4 | Glut4 | ACACTGGTCCTAGCTGTATTCT | CCAGCCACGTTGCATTGTA |  |
|  | ADIPOR1 | AdipoR1 | ACGTTGGAGAGTCATCCCGTAT | CTCTGTGTGGATGCGGAAGAT |  |
|  | B2M | B2M | TTCTGGTGCTTGTCTCACTGA | CAGTATGTTCGGCTTCCCATTC |  |
|  |  |  |  |  |  |
